# Understanding the Decoupled Effects of Cations and Anions Doping for High-Performance Perovskite Solar Cells

**DOI:** 10.1007/s40820-025-01655-x

**Published:** 2025-02-14

**Authors:** Tianxiang Hu, Yixi Wang, Kai Liu, Jia Liu, Haoyang Zhang, Qudrat Ullah Khan, Shijie Dai, Weifan Qian, Ruochen Liu, Yanyan Wang, Chongyuan Li, Zhenru Zhang, Mingxiang Luo, Xiaofei Yue, Chunxiao Cong, Yuan Yongbo, Anran Yu, Jia Zhang, Yiqiang Zhan

**Affiliations:** 1 Center of Micro-Nano System, School of Information Science and Technology, Fudan University, Shanghai, 200438 People’s Republic of China; 2 The State Key Laboratory of Photovoltaic Science and Technology, Institute of Optoelectronics, Fudan University, Shanghai, 200438 People’s Republic of China; 3 Institute for Electric Light Sources, School of Information Science and Technology, Fudan University, Shanghai, 200438 People’s Republic of China; 4 Hunan Key Laboratory of Nanophotonics and Devices, School of Physics, Central South University, Changsha, 410083 People’s Republic of China; 5 Vanced Materials Technology (Zhongshan) Co., Ltd., Guangdong, 528437 People’s Republic of China

**Keywords:** Perovskite solar cells, Interstitial doping, Effect decoupling, Ion migration

## Abstract

The past decade has witnessed the rapid increasement in power conversion efficiency of perovskite solar cells (PSCs). However, serious ion migration hampers their operational stability. Although dopants composed of varied cations and anions are introduced into perovskite to suppress ion migration, the impact of cations or anions is not individually explored, which hinders the evaluation of different cations and further application of doping strategy. Here we report that a special group of sulfonic anions (like CF_3_SO_3_^−^) successfully introduce alkaline earth ions (like Ca^2+^) into perovskite lattice compared to its halide counterparts. Furthermore, with effective crystallization regulation and defect passivation of sulfonic anions, perovskite with Ca(CF_3_SO_3_)_2_ shows reduced PbI_2_ residue and metallic Pb^0^ defects; thereby, corresponding PSCs show an enhanced PCE of 24.95%. Finally by comparing the properties of perovskite with Ca(CF_3_SO_3_)_2_ and FACF_3_SO_3_, we found that doped Ca^2+^ significantly suppressed halide migration with an activation energy of 1.246 eV which accounts for the improved operational stability of Ca(CF_3_SO_3_)_2_-doped PSCs, while no obvious impact of Ca^2+^on trap density is observed. Combining the benefits of cations and anions, this study presents an effective method to decouple the effects of cations and anions and fabricate efficient and stable PSCs.

**Supplementary Information:**

The online version contains supplementary material available at 10.1007/s40820-025-01655-x.

## Highlights

- Alkaline earth cations are successfully incorporated into perovskite lattice with the aid of sulfonic acid anions, while alkaline earth metal halides are lack of doping capacity.
- The sulfonic acid anions effectively regulate the crystallization of perovskite and passivate the metallic Pb_0_ defect states, thereby improving the power conversion efficiency of perovskite solar cells.
- By comparing the property of FACF_3_SO_3_ and Ca(CF_3_SO_3_)_2_-doped perovskite films, the impact of suppressing halide migration with an activation energy of 1.246 eV is attributed to Ca_2+_ cations, thus providing methodology for decoupling the effects of cations and anions.

**Supplementary Information:**

The online version contains supplementary material available at 10.1007/s40820-025-01655-x.

## Graphical abstract

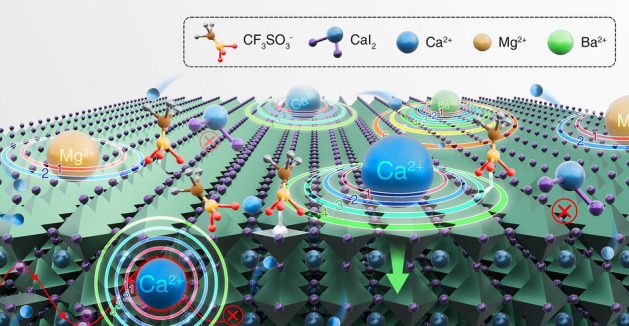

## Introduction

In the past decade, metal halide perovskite solar cells have experienced significant increase in power conversion efficiency of up to 26.1%, which is comparable to their inorganic mono-silicon counterpart, offering promising potential for future commercialization [1, 2]. Despite the excellent optoelectronic properties and low manufacturing costs, the inferior operational stability of perovskite solar cells (PSCs) under working environment is one of the main reasons that undermines their potential commercialization [3–7]. Among the degradation origins of perovskite photovoltaic devices, the halide migration of perovskite photoactive layer under electric field, light or heat has been recognized as a vital cause for deterioration, leading to bulky defects and further decomposition of perovskites [8–15]. Hence, inhibiting the displacement of halide ions during the operation process is crucial for improving the durability of PSCs.

Interstitial incorporation of alkali metal ions has been widely used to suppress the ion migration of perovskites. Alkali halides such as cesium iodide (CsI), potassium iodide (KI), sodium iodide (NaI) and rubidium iodide (RbI) have been frequently reported to reduce the current–voltage hysteresis and prolong the life span of PSCs [16–18]. Recently, doping cations with higher valence states like calcium (Ca^2+^), barium (Ba^2+^) and neodymium (Nd^3+^) ions have been reported to be more effective in mitigating the halide migration with less dopant dosage and smaller lattice distortion, indicating great potential for enhancing the lifetime of PSCs in practical applications [19]. In previous attempts to introduce doping cations with higher valence states, different ionic compounds with varied types of anions such as halide ions (Cl^−^, I^−^) and organic acid anions are added into perovskite precursors [19]. However, the anions themselves can affect the composition and crystallization process of perovskite films through halide substitution [20, 21], or Lewis coordination [22, 23], which makes it complicated to understand the doping effects of ionic compounds on the efficiency and stability of PSCs. What’s more, different anions can also influence the doping efficiency of the cations, which has not yet been systematically explored. Therefore, it is crucial to explore and decouple the effects of doped interstitial cations and anions to optimize the doping strategy toward highly efficient and stable PSCs.

Aiming at these points, we tried to add some dopants containing different anions (such as iodide (I^−^) and trifluoromethanesulfonate (CF_3_SO_3_^−^)) and divalent metal ions (such as magnesium (Mg^2+^), calcium (Ca^2+^) and barium (Ba^2+^)) into lead iodide solution to fabricate perovskite film with typical two-step process. As a result, we found that it’s very hard to dope the divalent metal cations like Ca^2+^ into perovskite when combining them with halide anions, while the highly polaric anions such as trifluoromethanesulfonate (CF_3_SO_3_^−^) and dobesilate (C_6_H_5_O_5_S^−^) composed of an electron-drawing group and a sulfonic group can largely promote the cation doping efficiency. The obvious peak shifts from X-ray diffraction (XRD) results clearly show that all divalent metal ions (Mg^2+^, Ca^2+^, and Ba^2+^) are successfully doped into the interstitial sites of the perovskite crystal lattices. Through systematically comparing the optoelectronic properties of the ionic compounds (Ca(CF_3_SO_3_)_2_ and formamidinium trifluoromethanesulfonate, FACF_3_SO_3_)-doped perovskite films and non-doped ones, we successfully decoupled the doping effects of anions and cations: (i) The doped anions such as CF_3_SO_3_^−^ show significant enhancement in crystallization and defect passivation of perovskite films evidenced by reduced PbI_2_ residuals and suppressed non-radiative recombination. The underlying reasons might be that the doped CF_3_SO_3_^−^ anions can induce a much more porous PbI_2_ film to facilitate the second step growth of perovskites, and also passivate the metallic Pb defects through efficient Lewis acid–base action between CF_3_SO_3_^−^ anions and metallic Pb; (ii) the doped cations like Ca^2+^ have ignorable impact on trap density of perovskite films but play an import role in inhibiting the halide migration. The temperature-dependent conductivity measurements shows that the activation energy of ion migration is enlarged from 0.420 to 1.246 eV, which is among the highest records [24–26]. Such efficient suppressing of ion migration could result from the reduced deep-level defects acting as ion migration pathways and the strong Coulomb interaction between the mobile halide ions and doped divalent metal cations located in the interstitial lattice sites [13, 19]. In addition, with favorable defect passivation and suppression of ion migration through ionic compounds doping method, the resulting PSCs exhibit a decent power conversion efficiency (PCE) of 24.95% and excellent operational stability, retaining over 90% of the maximum efficiency after MPP tracking for 1000 h under continuous illumination (100 mW cm^−2^).

## Experimental Section

### Materials

PbI_2_ (99.99%) and cyclohexylmethylammonium iodide (CHMAI) were purchased from Tokyo Chemical Industry (TCI, Japan). Mg(CF_3_SO_3_)_2_, Ca(CF_3_SO_3_)_2_, Ba(CF_3_SO_3_)_2_, Calcium dobesilate, lithium bis(trifluoromethanesulfonyl)imide (Li-TFSI) and Tris(2-(1H-pyrazol-1-yl)-4-tert-butylpyridine)-cobalt(III) Tris(bis(trifluoromethylsulfonyl)imide)) (Co-TFSI) were bought from Sigma-Aldrich. Formamidinium iodide (FAI), methylammonium iodide (MAI) were purchased from Greatcell Solar Materials. Methylammonium chloride (MACl) was bought from Materwin. SnO_2_ colloid solution (15 wt%) was brought from Alfa-aesar. 2,2',7,7'-Tetrakis[N,N-di(4-methoxyphenyl)amino]-9,9'-spirobifluorene (Spiro-OMeTAD) was purchased from Xi’an Polymer Light Technology. N,N-dimethylformamide (DMF, 99.8%), dimethyl sulfoxide (DMSO, 99.9%), isopropanol (IPA) (99.7%), chlorobenzene (CB 99.8%), 4-tert-butylpyridine (tBP), and acetonitrile (ACN, 99.9%) were also bought from Sigma-Aldrich.

### ***Synthesis of Formamidine Trifluoromethanesulfonate (CH(NH***_***2***_***)***_***2***_*** CF***_***3***_***SO***_***3***_***)***

For the synthesis of CH(NH_2_)_2_ CF_3_SO_3_, equimolar amount of formamidine diacetate and trifluoromethanesulfonic acid were placed in round bottom flask under the nitrogen atmosphere. Then 5 g of formamidine acetate was put into round bottom flask and the flask was transferred into the stand under 0 °C purged nitrogen for 10 min and later on starts the drop wise addition of trifluoromethanesulfonic acid under vigorous stirring. The temperature was firstly kept at 0 °C during the addition of acid and later on increased up to 85 °C for 2 h. Later on, the reaction medium was cooled to room temperature and evaporate the unreacted acid under reduced pressure to obtain the white powder of (CH(NH_2_)_2_ CF_3_SO_3_).

### Device Fabrication

SnO_2_ colloid solution was diluted with deionized water (1: 5, v: v) and stirred at room temperature for 1 h. The mixed aqueous solution was spin-coated onto the glass/FTO substrates at 3000 rpm for 30 s and then the substrates were annealed at 180 °C for 330 min in ambient air. To prepare the precursor solutions for the perovskite layers, 1.5 M PbI_2_ solutions (DMF/DMSO, v: v = 9: 1) with diffrent molar ratio of Ca(CF_3_SO_3_)_2_ (0, 0.15%, 0.25%, 0.4%, 1%) were stirred at 60 °C for 6 h, and organic ammonium salt solutions (FAI: MACl: MAI = 90: 9: 6.4 mg in 1 mL IPA) were stirred at room temperature for 6 h. In an N_2_-filled glove box, PbI_2_ solutions were spin-coated onto the deposited SnO_2_ layers at 1500 rpm for 30 s and immediately transferred to a hot plate for annealing at 70 °C for 1 min. After cooling to room temperature, 100 uL of organic ammonium salt solutions were rapidly poured down and spin-coated at 2000 rpm for 30 s. The substrates were transferred to a hot table in ambient air (30%–40% relative humidity) to anneal at 150 °C for 15 min. For the passivation treatment, a CHMAI solution (6 mg in 1 mL IPA) was deposited on the cooled-down perovskite films at 6000 rpm for 30 s without further annealing in an N_2_-filled glove box. A stock spiro-OMeTAD solution consisting of 72.3 mg in 1 mL of CB with 18 uL of Li-TFSI solution (520 mg of Li-TFSI powder in 1 mL of ACN), 29 uL of Co-TFSI solution (300 mg of Co-TFSI powder in 1 mL of ACN) 29 uL of tBP was then coated on the modified-perovskite films at 3000 rpm for 30 s. Finally, 85 nm of Au electrode was thermally evaporated at 2 × 10^–4^ Pa.

### Characterizations

X-ray diffraction (XRD) measurements were carried out using X-ray diffractometer (D8 Advance) with a monochromatic Cu-Kα (λ = 1.5405 Å) X-ray source. Scanning electron microscopy (SEM) images of perovskite films were taken by Hitachi S-4800 and ZEISS SIGMA HD. Fourier transform infrared spectroscopy (FTIR) spectra were recorded by IR spectrometer instrument (BRUKER, VERTEX 70). X-ray photoemission spectroscopy (XPS) data were measured in an ultrahigh vacuum surface analysis system equipped with SCIENTA R3000 spectrometer with a base pressure of 10^−10^ mbar and with monochromatic Al Kα 1,486.6 eV source. All spectra ware calibrated by referring to Fermi level edge and Au 4*f*_7/2_ position of the Ar^+^ ion sputter-cleaned Au film.

The *J-V* curves of the PSCs were measured using a Keithley 2602B source in the N_2_-filled glove box at room temperature under AM 1.5 G condition at an intensity of 100 mW cm^−2^, calibrated by a standard Si solar cell (PVM937, Newport). The standard Si is calibrated every month. A 450-W xenon lamp (Oriel solar simulator, 94023A) was used as a light source. The active area of PSCs is 0.107 cm^2^ defined by the cross of patterned Au and FTO electrode and further calibrated by the microscope. The aperture area is 0.0865 cm^2^. The *J-V* curves were obtained both at forward scan (from − 0.2 to 1.2 V, step 20 mV) and reverse scan (from 1.2 to − 0.2 V, step 20 mV) without any pre-conditioning before the test. EQE data were acquired by an EQE system (Enli Tech, Taiwan) using 100 Hz chopped monochromatic light (300–900 nm). Transient photovoltage measurement system are conducted at Oriental Spectra Technology (Guangzhou) Co., Ltd. Operational stability was tested using white LED at 100 mW cm^−2^ without UV filter. Samples were under illumination in an N_2_ environment at 25 ± 5 °C without any encapsulation. Maximum power point (MPP) dynamic tracking test adopts disturbance observation tracking. We first set the MPPT test duration and then set the number of IV scans in the test (for example, if the test duration is 1,000 h and the number of IV scans are 40 times, IV scans will be performed every 25 h in the test). For each IV scan, V_max1_ of the current scan was captured as the disturbance base, and 25 step scans were performed at plus/minus 50 mV. V_max2_ within the recording interval was captured and multiplied by the current at that time to obtain a MPP, and a MPP was recorded according to the MPPT scanning interval. In this way, Vmax has been updated after each scan and the next perturbation scan is performed in the vicinity of Vmax. Mott-Schottky curves with capacitance–voltage measurements were performed by a ZAHNER PP211 electrochemical workstation at 10 kHz with bias voltages ranging from 1.2 to 0 V and an AC voltage of 20 mV was used to test the corresponding capacitance at shifty bias voltage. Activation energy measurement of ion migration was carried out by measuring the temperature-dependent electric conductivity of Au/perovskite/Au structure under an electric field of 0.35 V μm^−1^. The activation energy *E*_a_ can be extracted by fitting the raw data points to the Arrhenius equation [27].

## Results and Discussion

### Successful Interstitial Doping with Sulfonic Acid Salts

**Fig. 1:**
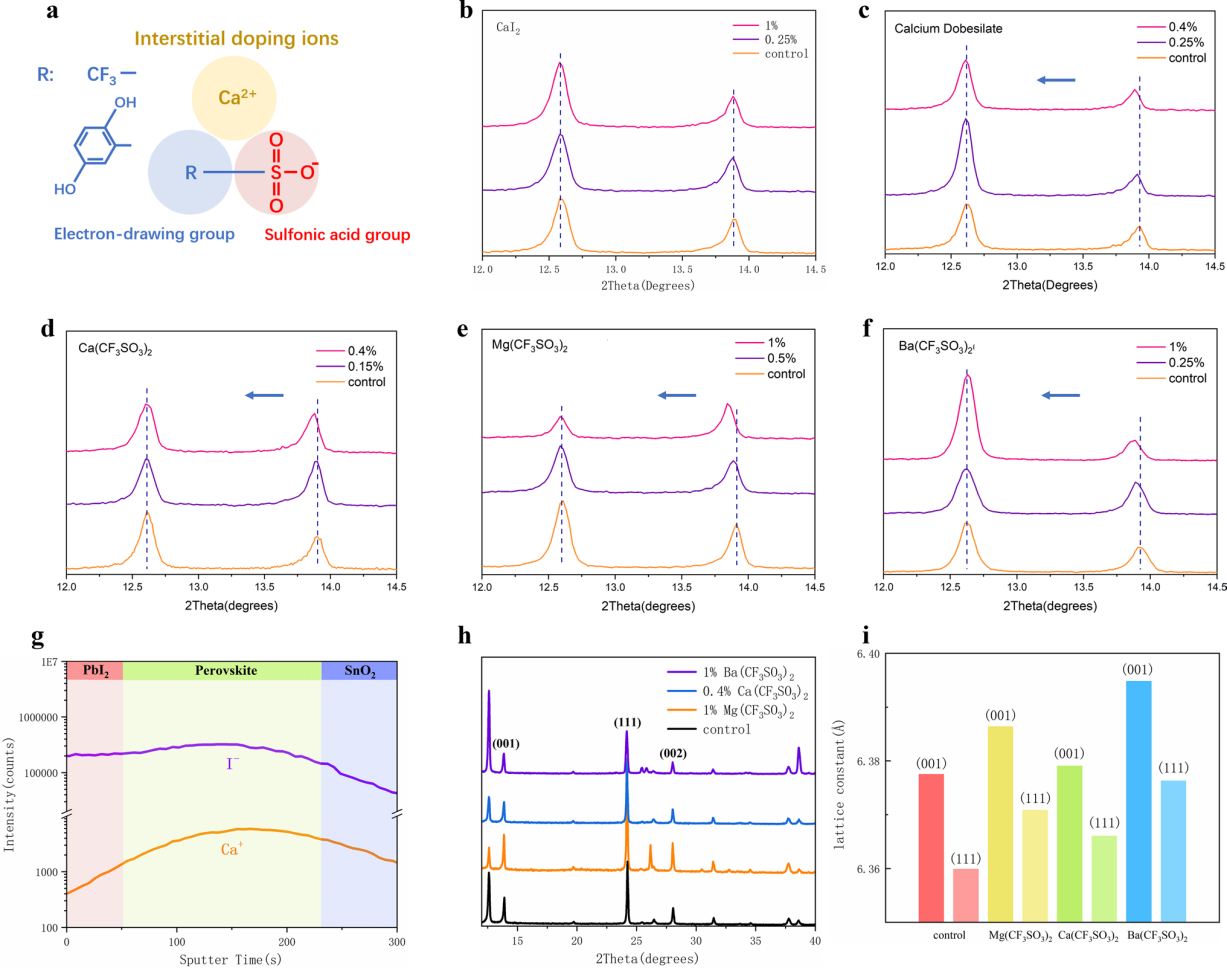
Interstitial doping with sulphonic acid strategy. **a** Molecular structure of the sulfonic acid dopants. Detailed XRD patterns of **b** CaI_2_, **c** calcium dobesilate, **d** Ca(CF_3_SO_3_)_2_, **e** Mg(CF_3_SO_3_)_2_ and **f** Ba(CF_3_SO_3_)_2_-doped perovskite films. **g** Ca^2+^ and I^−^distribution of 0.15% Ca(CF_3_SO_3_)_2_-doped perovskite measured by TOF–SIMS test. **h** Full XRD pattens of control, 1% Mg(CF_3_SO_3_)_2_-doped, 0.4% Ca(CF_3_SO_3_)_2_-doped and 1% Ba(CF_3_SO_3_)_2_-doped perovskite films. **i** Calculated lattice constants of control, 1% Mg(CF_3_SO_3_)_2_-doped, 0.4% Ca(CF_3_SO_3_)_2_-doped and 1% Ba(CF_3_SO_3_)_2_-doped perovskite

To probe the varied effects of different anions on the cation insertion into perovskite lattice, we added diverse dopants to PbI_2_ solution and fabricated perovskite films according to a typical two-step process. Among the dopants we have introduced, a special group with specific structure as illustrated in Fig. 1a caught our attention. These dopants are made up of interstitial doping cations and anions that contain an electron-drawing group (hydroquinone or trifluoromethyl) and a sulfonic acid group. To confirm whether the dopants are successfully incorporated into perovskite lattice, XRD measurements were carried out for perovskite films with various dopants and doping concentrations. When choosing the doping cations, we firstly choose Ca^2+^ with an ionic radius of 100 pm, which is slightly smaller than that of Pb^2+^ and would help to determine the doping sites. While substituting Pb^2+^ with Ca^2+^ will lead to lattice contraction, interstitial doping will bring about lattice expansion. To exclude the impact of halides, CaI_2_ was utilized as a representative for halogenides in our FAPbI_3_ dominated system. As shown in Fig. 1b, the XRD results of perovskite films with 0%, 0.25%, and 1% molar ratio of CaI_2_ additives show distinct α-FAPbI_3_ peaks and PbI_2_ residue peaks. All the films with different amount of CaI_2_ exhibit identical (001) peak of α-FAPbI_3_ at around 14 degrees of 2θ (where θ is the Bragg angle), indicating that Ca^2+^ ions have not been successfully doped into the lattice. By contrast, for the perovskite films with dopants containing highly polaric anions, the XRD peaks show obvious shift to lower 2θ angle. In Fig. 1c (Ca(C_6_H_5_O_5_S)_2_) and Fig. 1d (Ca(CF_3_SO_3_)_2_), the (001) peak of perovskite gradually shift to smaller 2θ position with the increment of doping amount, indicating lattice volume expansion and successful interstitial doping of Ca^2+^ [19].

As the CF_3_SO_3_^−^-doped perovskites show better crystallinity with less PbI_2_ residue compared to the calcium dobesilate incorporated ones, this anion was chosen as prototype for more in-depth study. To further verify whether the highly polaric anions could promote the cation doping efficiency, we compared the XRD characterizations for MgI_2_, BaI_2_ and Mg(CF_3_SO_3_)_2_, Ba(CF_3_SO_3_)_2_ incorporated perovskite films, which showed an identical trend. In Fig. 1d, e, with the aid of CF_3_SO_3_^−^ anion, the Mg^2+^ and Ba^2+^ were also successfully doped into perovskites evidenced by the similar peak shifts of α-FAPbI_3_ (001) phase to lower 2θ value. While identical XRD peaks (Fig. S2a, b) were observed in the MgI_2_, BaI_2_-doped samples, implying no lattice dilation. To investigate the distribution of doping cations in the perovskite layer, we firstly performed top-surface EDS mapping for Mg(CF_3_SO_3_)_2_, Ca(CF_3_SO_3_)_2_, and Ba(CF_3_SO_3_)_2_-doped perovskite films, respectively. As shown in Fig. S1, all the doping cations exhibit uniform distribution within in the horizontal plane. We further conducted TOF–SIMS test for Ca(CF_3_SO_3_)_2_-doped perovskite to explore depth-dependent doping cation distribution (Fig. 1g), the intensity of Ca^+^ cation keeps a nearly constant ratio to I^−^ in the perovskite layer, indicating the uniform distribution of Ca^2+^.

When dissolved in solvents like N, N-dimethylformamide (DMF), dimethyl sulfoxide (DMSO) which are commonly used to fabricate perovskite layer, the used metal halide dopants such as CaI_2_ exist in the form of colloids, where metal cations and halide anions are closely coupled. To explore whether the solvents can influence the dissociation of metal cations and halide anions thus hamper the doping process, we further dissolved CaI_2_ into water where cations and anions are fully decoupled. Then trace aqueous solution of CaI_2_ were added into PbI_2_ (5 uL CaI_2_ aqueous solution into 1 mL PbI_2_ solution) to fabricate perovskite. The XRD results of corresponding perovskite films are shown in Fig. S2c, where the main peak of α-FAPbI_3_ doesn’t show any shift for all doping concentrations. These results manifest that the ionized metal halides are still lack of doping capabilities, which may be caused by the large discrepancy between Ca^2+^ and Pb^2+^. By contrast, CF_3_SO_3_^−^ has strong interaction with PbI_2_ and could anchor Ca^2+^ to the inorganic framework of perovskite during the crystallization process, thus boost the metal cation doping efficiency [28].

From the above experimental results, with the aid of CF_3_SO_3_^−^ cations, all the doping cations have been inserted into perovskite lattice to occupy interstitial site revealing uniform doping pattern across the whole perovskite layer. To get a more quantitative view of the doped perovskite structure, we then calculated the lattice constants for control, 1% Mg(CF_3_SO_3_)_2_-doped, 0.4% Ca(CF_3_SO_3_)_2_-doped and 1% Ba(CF_3_SO_3_)_2_-doped perovskite films from the full XRD pattern in Fig. 1h. The detailed calculation method is depicted in supplementary Note 1, and the fitting results are given in supplementary Table S1. The extracted lattice constants using (001) and (111) diffraction peaks for varied doping cations are shown in Fig. 1i and Table S2. Though the results calculated from different crystal indices have slight variations, the tendency keeps the same. Compared to control perovskite, all the doped perovskites show enlarged lattice constants. As for the different doping cations using the same doping concentration, Ba^2+^ cations with larger ionic radii induce greater lattice expansion than Mg^2+^ cations, which strengthens our findings that these cations occupy interstitial sites in perovskite lattice.

### ***Crystallization and Morphology Regulation Impact of CF***_***3***_***SO***_***3***_^***−***^*** Anions***

**Fig. 2:**
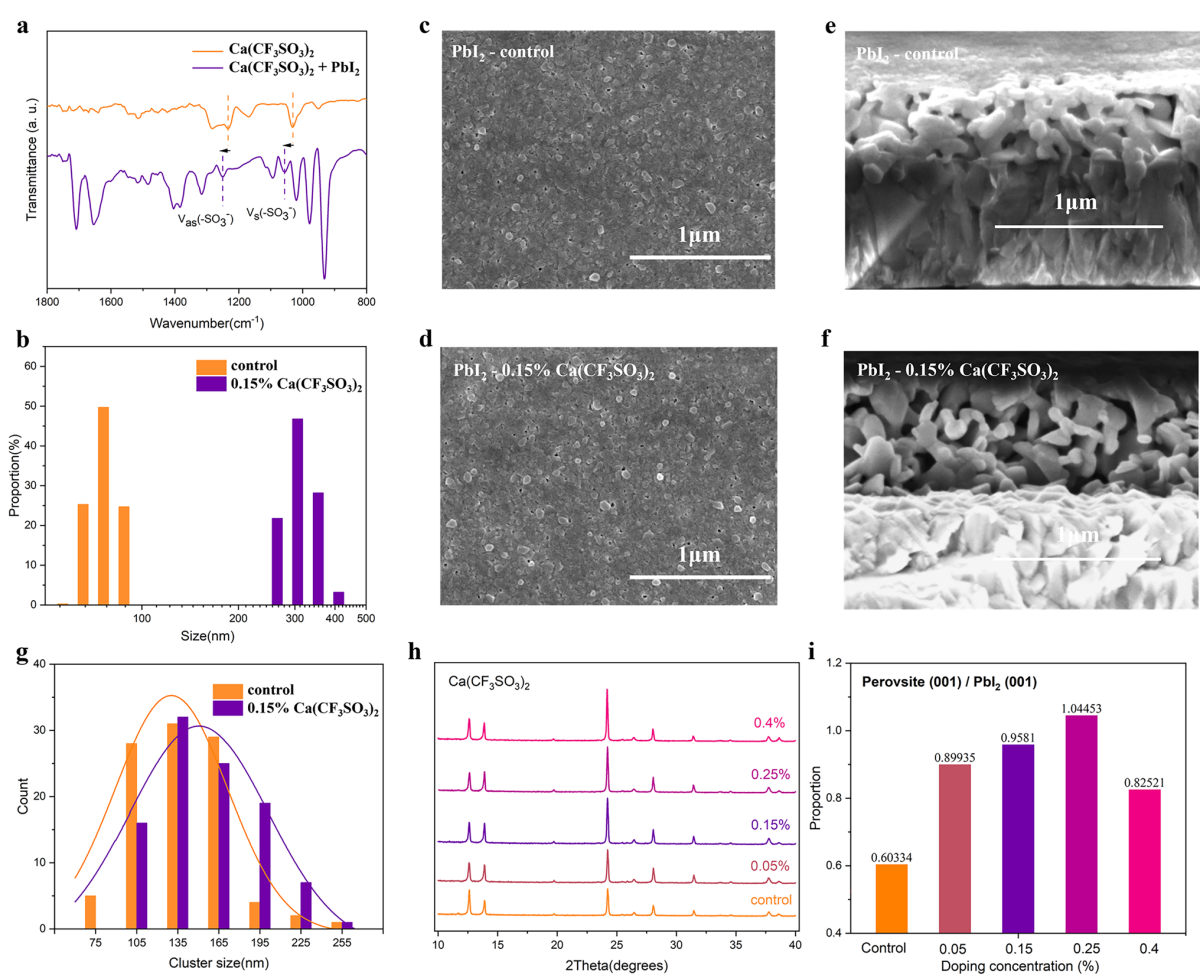
Characterizations of crystallizations of PbI_2_ and perovskite. **a** FTIR spectra of Ca(CF_3_SO_3_)_2_ before and after mixing with PbI_2_. **b** Dynamic light scattering spectra of PbI_2_ solution and PbI_2_ solution with 0.15% Ca(CF_3_SO_3_)_2_. Top-view SEM images of **c** PbI_2_ film and **d** PbI_2_ film with 0.15% Ca(CF_3_SO_3_)_2_. Cross-sectional SEM images of **e** PbI_2_ film and **f** PbI_2_ film with 0.15% Ca(CF_3_SO_3_)_2_. **g** Cluster size distribution in PbI_2_ and 0.15% Ca(CF_3_SO_3_)_2_ processed PbI_2_ film. **h** Full XRD patterns of fabricated perovskite films with 0%, 0.05%, 0.15%, 0.25% and 0.4% Ca(CF_3_SO_3_)_2_. **i** Ratio of perovskite (001) peak intensity to PbI_2_ (001) peak intensity in perovskite films with varied Ca(CF_3_SO_3_)_2_ concentrations

To understand the potential chemical interactions between Ca(CF_3_SO_3_)_2_ and PbI_2_, we conducted Fourier transform infrared spectroscopy (FTIR) characterizations of Ca(CF_3_SO_3_)_2_ before and after mixing with Ca(CF_3_SO_3_)_2_. As displayed in Fig. 2a, the peaks located at 1234.2 and 1032.2 cm^−1^ are attributed to symmetrical (ν_s_) and antisymmetric (ν_as_) stretching mode of sulphonic group in CF_3_SO_3_^−^, respectively [29]. For films of mixed Ca(CF_3_SO_3_)_2_ and PbI_2_, the corresponding stretching vibration peaks redshifted to higher wavenumbers of 1251.4 and 1056.4 cm^−1^, which is owing to the weakened spatial symmetry of -SO_3_^−^ after one oxygen atom forms coordination bonds with PbI_2_ [30, 31]. After confirming the coordination between CF_3_SO_3_^−^ anion and PbI_2_, we then compared the PbI_2_ solutions with and without Ca(CF_3_SO_3_)_2_ to check its effect on the PbI_2_ cluster size distribution. According to the dynamic light scattering (DLTS) statistics, the PbI_2_ clusters with Ca(CF_3_SO_3_)_2_ are markedly larger with an average size of more than 300 nm, while pristine colloidal PbI_2_ shows a mean diameter less than 100 nm (Fig. 2b). The larger cluster size could be attributed to the interaction between the sulfonic acid group in CF_3_SO_3_^−^ anion and Pb-I octahedra, which enables more PbI_2_ clusters to surpass the Gibbs free energy barrier directly and go through the nucleation process [32].

To understand the effect of CF_3_SO_3_^−^ anions on morphology and crystallinity of PbI_2_ and perovskite films, we further performed SEM measurements of PbI_2_ films with different amount of dopants. As shown in Fig. 2c, d, compared to the dense morphology of control film, the PbI_2_ film with 0.15% Ca(CF_3_SO_3_)_2_ addition exhibits a much more porous structure, which can facilitate the permeation of ammonium salts and the growth of perovskite grains [33–35]. The morphology of PbI_2_ films with other amounts of dopants in Fig. S3 show similar trend. From the cross-sectional SEM images in Fig. 2e, f, PbI_2_ film with Ca(CF_3_SO_3_)_2_ dopant also displays a much more porous morphology in the vertical direction. Considering the cluster size distribution in PbI_2_ colloid solution, we speculate that the porous structure of Ca(CF_3_SO_3_)_2_-doped film is from the preaggregation of clusters induced by strong coordination between CF_3_SO_3_^−^ anion and PbI_2_ [36]. We also analyzed the grain size distribution from the SEM images of PbI_2_ films. As shown in Fig. 2g, PbI_2_ films with Ca(CF_3_SO_3_)_2_ treatment reveal a larger average grain size and more grains with a diameter over 180 nm. Moreover, the XRD test is performed to understand the crystallinity of control and Ca(CF_3_SO_3_)_2_-doped PbI_2_ films (Fig. S4a). Compared to pristine PbI_2_ film, doped film shows a much stronger intensity of PbI_2_ (001) plane and reduced full width at half-maximum (FWHM) (Fig. S4b), exhibiting largely enhanced crystallinity. The lager crystal size and improved crystallinity of PbI_2_ can lower the nucleation free energy of perovskite, thus facilitates the subsequent growth of high-quality perovskite films [37, 38].

To further explore the effect of CF_3_SO_3_^−^ anions on the crystallinity of perovskite films, we carried out XRD characterizations for perovskite films with various Ca(CF_3_SO_3_)_2_ doping ratios(0%, 0.05%, 0.15%, 0.25%, 0.4%), respectively. The XRD pattern (Fig. 2j) demonstrates that compared to the control film, samples with dopants displays a stronger perovskite (001) peak and a smaller FWHM, indicating better crystallinity. What’s more, when we compare the ratio of perovskite (001) plane and PbI_2_ (001) plane in perovskite films with and without dopants (Fig. 2k), it is noted that the ratio drops tremendously for all doping concentrations. We attribute the enhanced crystallinity and reduced PbI_2_ residue of Ca(CF_3_SO_3_)_2_-doped perovskite films to better quality and porous structure of PbI_2_ films, which endows facile and sufficient permeation of ammonium salts and rapid conversion to perovskite phases [32].

**Fig. 3:**
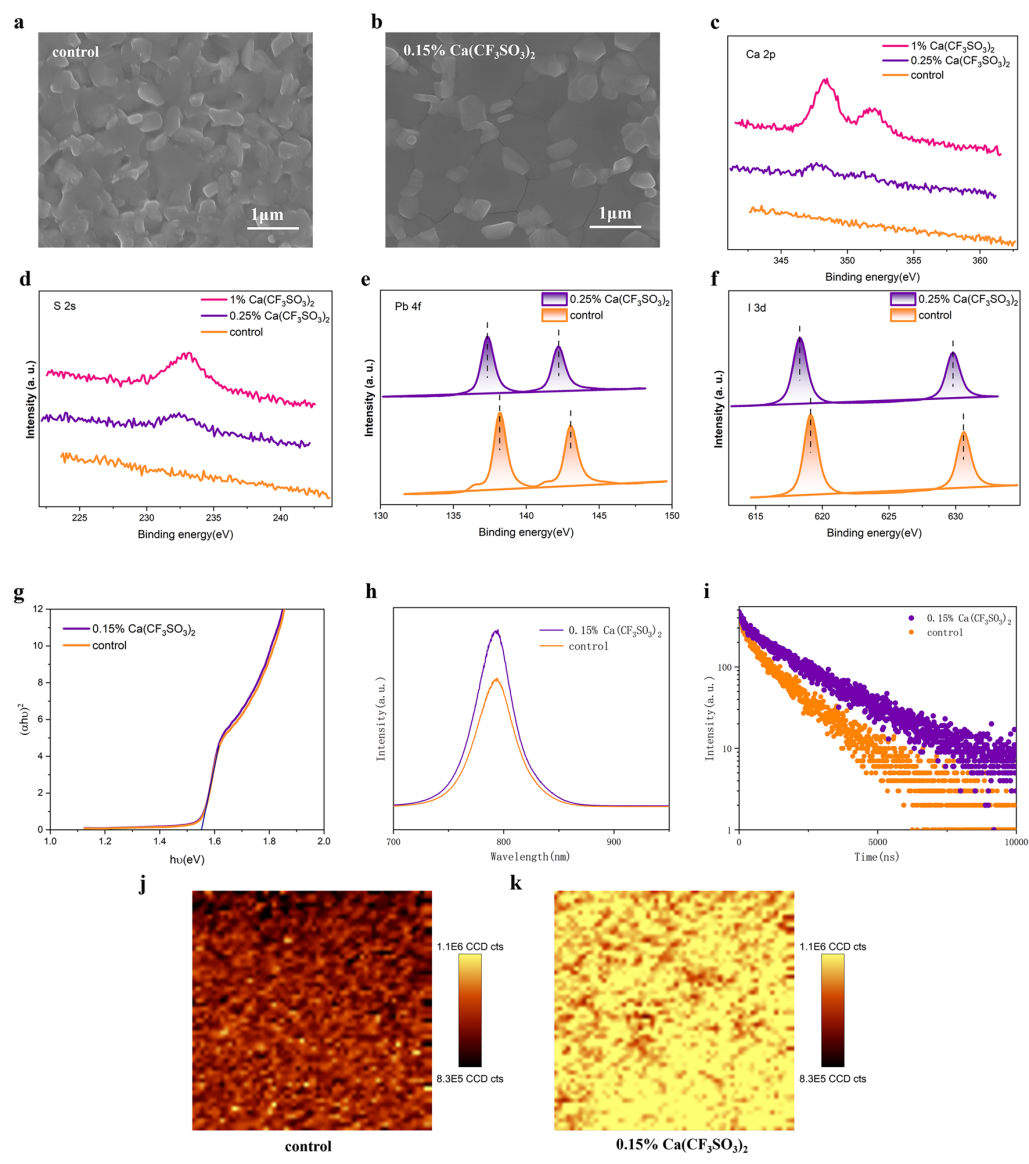
Characterizations and optoelectronic properties of perovskite films. Top-view SEM images of **a** control perovskite and **b** perovskite films with 0.15% Ca(CF_3_SO_3_)_2_. **c** Ca 2*p*, **d** S 2*s*, **e** Pb 4*f*, **f** I 3*d* XPS spectra of perovskite films doped with varied concentration of 0.15% Ca(CF_3_SO_3_)_2_. **g** Tauc plot of perovskite films with and without Ca(CF_3_SO_3_)_2_. **h** Steady-state PL and **i** TRPL spectra of perovskite films with and without Ca(CF_3_SO_3_)_2_ deposited on glass substrate. PL mapping images of **j** pristine perovskite film and **k** perovskite film with 0.15% Ca(CF_3_SO_3_)_2_

We further perform SEM measurements to verify the effect of CF_3_SO_3_^−^ anion on crystal growth and optical properties of perovskite thin films. As shown in Fig. 3a, owing to the chaotic crystallization caused by stiff intercalation of ammonium salts, control perovskite film consists of heterogenous and disordered grains covered by massive PbI_2_ residue. By contrast, the perovskite film with 0.15% Ca(CF_3_SO_3_)_2_ comprises significantly larger and compact grains with vastly reduced PbI_2_ remnants, showing a more adequate transition into perovskite with enhanced crystallinity, in agreement with the XRD results. To exclude the latent effect of Ca^2+^ cations on the morphology of perovskite, we also performed SEM measurements of perovskite with varied amount of formamidine trifluoromethanesulfonate (FACF_3_SO_3_) additive (0%, 0.3%, 0.5%, 0.8%), respectively (Fig. S5). We then performed cross-sectional SEM to explore the morphology of perovskite films in the vertical direction. As shown in Fig. S6a, control perovskite film exhibited piled grains with disordered orientation, while Ca-doped perovskite demonstrated much larger grains perpendicular to the substrate, which benefits the charge transport to the adjacent layers (Fig. S6b). For all perovskite film with FACF_3_SO_3_ additive, in consistence with the tendency shown in Ca(CF_3_SO_3_)_2_-doped films, the grain size increased a lot and less PbI_2_ could be observed, especially for high additive concentrations. Hence the improvement in morphology and crystallinity of perovskite films should be attributed to CF_3_SO_3_^−^ anions. The SEM results confirms the facilitation effect of CF_3_SO_3_^−^ anion on perovskite crystal growth owing to PbI_2_ film with better crystallinity and lower Gibbs free energy, leading to adequate interaction and enlarged grain sizes [32].

### ***Defect Passivation Effect of CF***_***3***_***SO***_***3***_^***−***^*** Anions and Photoelectric Properties of Perovskite Films***

To further understand the chemical reaction between CF_3_SO_3_^−^ anion and perovskite, XPS was employed for Ca(CF_3_SO_3_)-doped perovskite films. As characteristic elements, Ca 2*p* and S 2*s* peaks for perovskite films with different doping concentrations are observed in Fig. 3c, d. With enlarged doping ratio, stronger peaks of Ca 2*p* and S 2*s* peaks emerged, proving successful incorporation of Ca(CF_3_SO_3_)_2_ into the perovskite films. The detailed Pb 4*f* and I 3*d* spectrum are displayed in Fig. 3e, f. In the Pb 4*f* sepectrum, in comformity with the FTIR results, Pb 4*f*_5/2_ and 4*f*_7/2_ peaks at 143.00 and 138.26 eV in pristine perovskite films slighty shifted to lower binding energy in Ca(CF_3_SO_3_)_2_-doped films, which could be attributed to the interaction between Pb and the sulfonic group (-SO_3_) [29, 30]. Moreover, two shoulder peaks at lower binding energy position of Pb 4*f*_5/2_ and 4*f*_7/2_ peaks in the control films are related to metallic Pb^0^, which may serve as deep level defects and non-radiactive recombination center, thus can inhibit the efficiency and long-term stability of perovskite solar cells [39]. In Ca(CF_3_SO_3_)_2_-doped films, the lone electron pairs of oxygen atoms belonging to the sulfonic group form strong coordination bonding with the empty Pb 6*p* orbitals, thus efficiently passivate the Pb^0^ defects, as can be perceived from the negligible shoulder peak in Fig. 3e. In I 3*d* spectrum (Fig. 3f), I 3*d*_3/2_ and I 3*d*_5/2_ peaks showed a downshift as well, which may be caused by the enlarged Pb-I bond distance due to the interstitial doping of Ca^2+^.

We then investigated the optical properties of perovskite films using ultraviolet–visible (UV–vis) absorption spectrum (Fig. S7). The perovskite film with 0.15% Ca(CF_3_SO_3_)_2_ shows lightly enhanced absorption due to better crystallinity. Additionally, 0.15% Ca(CF_3_SO_3_)_2_-doped perovskite and control perovskite exhibited identical optical bandgap of 1.53 eV according to the Tauc plot (Fig. 3g). Thus, the minor doping ratio of 0.15% would not observably alter the absorption characteristics of FAPbI_3_. We further performed the steady-state photoluminescence (PL) and time-resolved photoluminescence (TRPL) measurements to unveil the non-radiative carrier recombination in different perovskite films. 0.15% Ca(CF_3_SO_3_)_2_-doped film demonstrated much stronger PL emission (Fig. 3h) and prolonged PL decay lifetime, indicating significantly restrained non-radiative recombination. To examine the uniformity of perovskite films, we further conducted confocal PL mapping test. As shown in Fig. 3j, k, perovskite film with 0.15% Ca(CF_3_SO_3_)_2_ revealed brighter PL signal with better homogeneity, indicating effectively passivated defects and regulated crystal growth.

### ***Photovoltaic Performance of Ca(CF***_***3***_***SO***_***3***_***)***_***2***_*** Doped Devices***

**Fig. 4:**
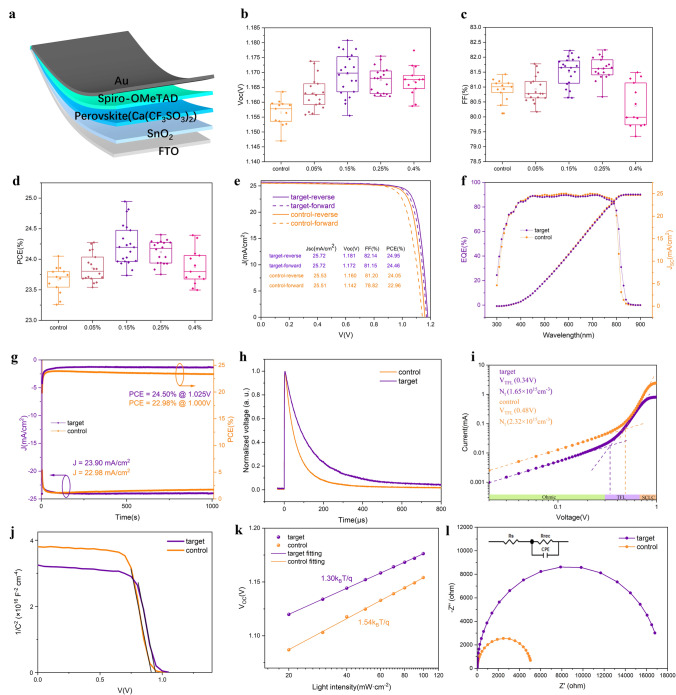
Photovoltaic performance of PSCs. **a** Device structure of fabricated perovskite solar cells. **b** V_OC_, **c** FF and **d** PCE statistical distribution of PSCs with different doping concentration of Ca(CF_3_SO_3_)_2_. **e** Forward and reverse scan *J-V* curves of best-performing target and control PSCs. **f** EQE spectra and corresponding integrated current of best-performing target and control PSCs. **g** SPO curves of best-performing target and control PSCs within 1000 s. **h** Normalized TPV spectra of best-performing target and control PSCs. **i** SCLC measurements of electron-only device (glass/FTO/SnO_2_/perovskite /PC_61_BM/Au) containing different perovskite films. **j** Mott-Schottky plots of different PSCs. **k** Light intensity dependent V_OC_ spectra of different PSCs. **l** EIS test of target and control PSCs

Perovskite photovoltaic devices with configuration of fluorine tin oxide (FTO)/ tin oxide (SnO_2_)/ perovskite/ Cyclohexylmethylammonium iodide (CHMAI)/ 2,2’,7,7’-tetrakis(N,N-dipmethoxyphenylamine)-9,9’-spirobifluorene (spiro-OMeTAD)/Au as illustrated in Fig. 4a were fabricated. We firstly tuned the concentration to explore the influence of Ca(CF_3_SO_3_)_2_ doping ratio on the device performance parameters like *J*_SC_, *V*_OC_, FF, and PCE to obtain the statistical distributions. As shown in Fig. S8, the average value of short-circuit current densities (*J*_SC_) of all doping concentrations did not show noteworthy variations with introducing the Ca(CF_3_SO_3_)_2_, while both the open-circuit voltage (*V*_OC_) and fill-factor (FF) were significantly enlarged, as demonstrated in Fig. 4b, c. Compared to the control devices, the average *V*_OC_ and FF of the best-performing doping concentration 0.15% significantly increased from 1.158 to 1.170 V and 80.92% to 81.65%, leading to an average PCE increment (Fig. 4d). For the sake of simplicity, the doping concentration below is defaulted to 0.15%, denoted as target, while pristine devices are called control. The results of reverse and forward sweeps of best-performing target and control devices are shown in Fig. 4e. The champion Ca(CF_3_SO_3_)_2_-doped device showed a PCE of 24.95% with a largely improved *V*_OC_ of 1.181 V and an FF of 82.14%, by contrast the control device only demonstrated an efficiency of 24.05%.

It is also noteworthy that target device showed a significantly reduced hysteresis with a PCE of 24.46% measured by forward sweep. According to the external quantum efficiency (EQE) results in Fig. 4f, the integrated current density of control and target devices are 24.70 and 24.83 mA cm^−2^, which coincide well with the *J*_SC_ value from *J*-*V* curves. We further performed steady power output (SPO) test (Fig. 4g) for control and Ca(CF_3_SO_3_)_2_-doped devices. Target devices demonstrated a stabilized PCE of 24.50% at the bias voltage of 1.025 V for 1000 s, while PCE of control device gradually dropped from 23.86% to 22.98% during the process.

To determine the trap density of states (*N*_t_) and carrier dynamics for different samples, we then conducted transient photovoltage (TPV) and space-charge-limited current (SCLC) characterizations. The TPV results illustrated in Fig. 4h verifies the prolonged carrier decay lifetime and suppressed non-radiative recombination of target device, in accordance with the TRPL results [40]. The dark *J*-*V* curves of electron-only devices (glass/FTO/SnO_2_/perovskite/PC_61_BM/Au) are shown in Fig. 4i. We calculated *N*_t_ of the control and target devices via Eq. (1) [29]:

1 $${{\varvec{N}}}_{{\varvec{t}}}=\boldsymbol{ }\frac{2{{\varvec{\varepsilon}}}_{0}{{\varvec{\varepsilon}}}_{{\varvec{r}}}{{\varvec{V}}}_{{\varvec{T}}{\varvec{F}}{\varvec{L}}}}{{\varvec{q}}{{\varvec{L}}}^{2}}$$

Here, ε_0_ stands for the vacuum permittivity (8.85 × 10^−12^ F m^−1^), q for the elemental charge, ε_r_ for the relative dielectric constant of perovskite (ε_r_ = 35) and L for the thickness of the perovskite absorber. According to the trap-filled limited voltage (*V*_TFL_) obtained from Fig. 4j, the derived *N*_t_ for control device is 2.32 × 10^15^ cm^−3^, whereas the derived *Nt* of target device is only 1.62 × 10^15^ cm^−3^. The reduced trap density is owing to better crystallization and effective defect passivation of Ca(CF_3_SO_3_)_2_-incorporated perovskites. To better understand the charge separation and extraction process of different devices, we further performed capacitance–voltage (*C*-*V*) measurements and employed Mott-Schottky analysis. The built-in electric fields of control and target device were estimated by Eq. (2):

2 $$\frac{1}{{{\varvec{C}}}^{2}}=\boldsymbol{ }\frac{2({{\varvec{V}}}_{{\varvec{b}}{\varvec{i}}}-{\varvec{V}})}{{{\varvec{\varepsilon}}}_{0}{{\varvec{\varepsilon}}}_{{\varvec{r}}}{\varvec{N}}{{\varvec{A}}}^{2}{\varvec{q}}}$$

Here A refers to the active area; *C* refers to the capacitance; N is the doping profile and *V* is the applied voltage [41]. From the Mott-Schottky results in Fig. 4k, the cross point of tangent line with the horizontal axis determines the built-in potential (*V*_bi_). The target device revealed a larger *V*_bi_ (0.95 V) than that of control device (0.91 V), which serves enhanced deriving force for better charge separation and extraction. We then tested the *V*_OC_ of different devices with varied light intensities. *V*_OC_ is loglinearly proportional to light intensity described by Eq. (3) [42]:

3 $${{\varvec{V}}}_{{\varvec{O}}{\varvec{C}}}\boldsymbol{ }\propto {\varvec{n}}\frac{{{\varvec{k}}}_{{\varvec{B}}}{\varvec{T}}}{{\varvec{q}}}\mathbf{ln}\left({{\varvec{P}}}_{0}\right)$$

where k_B_ is Boltzmann's constant, T is the absolute thermodynamic temperature and n is the ideality factor. The slope nk_B_T q^−1^ reflects the carrier recombination kinetics in PSCs. As derived from Fig. 4k, the target device exhibits a smaller slope (1.30 k_B_T q^−1^) than the control device (1.54 k_B_T q^−1^), which reveals the significantly suppressed trap-assisted non-radiative recombination in Ca(CF_3_SO_3_)_2_-doped devices. To further clarify the interfacial charge transfer and recombination process, we performed electrochemical impedance spectroscopy (EIS) and the results are shown in Fig. 4k. The charge transfer resistance (*R*_S_) and recombination resistance (*R*_rec_) of control and target devices were fitted with the illustrated equivalent circuit. We found that the target device has a smaller *R*_S_ and an enlarged *R*_rec_, indicating the improved charge extraction and suppressed non-radiative recombination.

### Decoupling the Effects of Anions and Interstitial Doping Cations

**Fig. 5:**
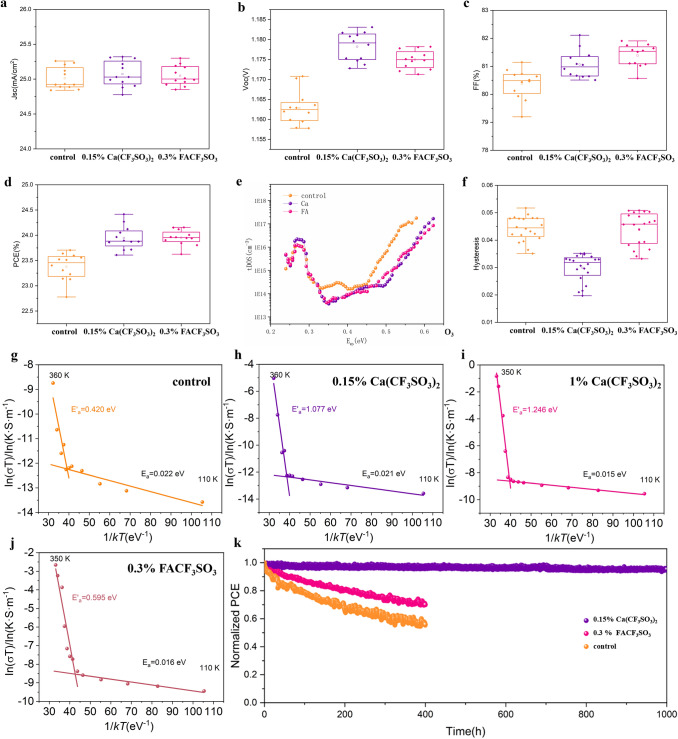
Decoupling the effect of cations and anions. **a** *J*_SC_, **b** *V*_OC_, **c** FF, and **d** PCE statistical distribution of control, FACF_3_SO_3_ processed and Ca(CF_3_SO_3_)_2_-doped PSCs. **e** Defect density of states of different trap depth. **f** Statistical hysteresis distribution of different PSCs. Temperature dependent conductivity measurements of lateral devices (Au/perovskite/Au) for **g** control perovskite, **h** perovskite with 0.15% Ca(CF_3_SO_3_)_2_ dopant, **i** perovskite with 1% Ca(CF_3_SO_3_)_2_ dopant and **j** 0.3% FACF_3_SO_3_ additive. **k** Operational stability results of the unencapsulated control, FACF_3_SO_3_ processed and Ca(CF_3_SO_3_)_2_-doped PSCs under continuous maximum power point (MPP) tracking at the temperature of 25 ± 5 °C under one sun equivalent white light light-emitting diode (LED) illumination

To gain preliminary understanding on the impact of doped Ca^2+^ cations and decouple them from the effects of anions, we carefully excluded the aforementioned effect of CF_3_SO_3_^−^ anions by introducing equivalent amount of FACF_3_SO_3_ into the system and compared device performance of Ca(CF_3_SO_3_)_2_-doped PSCs and devices with FACF_3_SO_3_ additive. Firstly, we fabricated PSCs with 0.15% Ca(CF_3_SO_3_)_2_ dopant, 0.3% FACF_3_SO_3_ additives and control devices to assess the role of doped Ca^2+^ in altering device performance. The detailed performance parameters *J*_SC_, *V*_OC_, FF, and PCE are shown in Fig. 5a–d, respectively. It is evident that compared to the control devices, 0.15% Ca(CF_3_SO_3_)_2_-doped PSCs and devices with 0.3% FACF_3_SO_3_ additives show obvious enhancements in both *V*_OC_ and FF and the improvements of PCE were in similar extent. Analogous PCE enhancement should be attributed to the crystallization regulation and defect passivation related to CF_3_SO_3_^−^ anions, as verified by the aforementioned SEM and XPS results. We further calculated the defect density in perovskite with Ca(CF_3_SO_3_)_2_ dopant and FACF_3_SO_3_ additive according to previously reported methods [43], as depicted in Fig. 5f. Both Ca(CF_3_SO_3_)_2_-doped devices and devices with 0.15% FACF_3_SO_3_ additives show significantly restrained deep-level trap states (beyond 0.4 eV) with defect density reduction of approximately one magnitude, which could be related to metallic Pb^0^ according to the energy level [39]. These data prove that a proper doping concentration of Ca^2+^ has no obvious impact on defect density and SRH recombination rate, thus negligible distinction was observed after eliminating the contribution of anions. To compare the effect of other doping cations on device performance, we also fabricated devices with 0.15% Mg(CF_3_SO_3_)_2_ and Ba(CF_3_SO_3_)_2_ dopants and the performance parameter distributions are shown in Fig. S9. The Mg^2+^-doped devices show slightly lower efficiency than Ca^2+^-doped ones. We speculate this is due to the smaller radii and relative mass of Mg^2+^, which may lead to migration of Mg^2+^ and impaired built-in electric field. We also noticed that Ba^2+^-doped devices exhibit inferior PCE, which can be attributed to the overlarge ionic radius of Ba^2+^ and thereby induced serious lattice distortion.

Despite bare improvement of PCE could be related to Ca^2+^ cations, we noticed that for best-performing devices with Ca(CF_3_SO_3_)_2_ dopants, the hysteresis has been obviously eliminated. To gain understanding into the origin of this phenomenon, we applied identical method and fabricated PSCs with 0.15% Ca(CF_3_SO_3_)_2_ dopant*,* 0.3% FACF_3_SO_3_ additives and control devices then investigated the statistical distribution of corresponding device hysteresis.

As shown in Fig. 5f, in contrast to the control samples, Ca^2+^-doped devices exhibited much smaller hysteresis index while the mean hysteresis of FACF_3_SO_3_ processed devices is quite close to that of control group. These results demonstrate that the reduced hysteresis is directly related to the trace doping of interstitial cations. To figure out the underlying mechanism, we performed temperature-dependent conductivity measurements for the lateral-structure devices (Au/perovskite (100 μm)/Au), as shown in Fig. 5g–j for control, 0.15% Ca(CF_3_SO_3_)_2_, 1% Ca(CF_3_SO_3_)_2_-doped PSCs and devices with 0.3% FACF_3_SO_3_ additives, respectively. The activation energy (*E*_a_) of different perovskites were extracted by fitting the data points measured at relatively higher temperatures based on Arrhenius equation [27]. Control perovskite exhibits an activation energy of 0.420 eV, while that of 0.15% Ca(CF_3_SO_3_)_2_-doped film is significantly enlarged to 1.077 eV and further increased to 1.246 eV with higher doping concentration (1%), which is among the highest value reported hitherto [24–26]. Perovskite with 0.3% FA additive demonstrated an slightly enlarged *E*_a_ of 0.595 eV, which could be attributed to decreased defect density and thus lessened migration pathways [44]. The migration activation energy of 0.15% Mg(CF_3_SO_3_)_2_ and Ba(CF_3_SO_3_)_2_-doped perovskite are illustrated in Fig. S10. The Mg^2+^-doped perovskite shows a slightly increased activation energy of 0.453 V, which indicates Mg^2+^ with a smaller relative mass are not sufficient to suppress the motion of iodide anions. Whereas Ba^2+^-doped perovskite exhibits a higher activation energy of 0.671 eV, which is still lower when compared to that of Ca^2+^-doped perovskite. We speculate it is due to the large lattice distortion and relative higher trap density induced by the insertion of oversize Ba^2+^ cations. By comparing the result of perovskite films with Ca(CF_3_SO_3_)_2_ dopant and FACF_3_SO_3_ additive, it is obvious that the interstitial doping ions play a vital role in suppressing ion migration with the aid of Coulomb interaction between Ca^2+^ and halide anions [19], accompanied by the contribution of reduced migration channels due to effective passivation of CF_3_SO_3_^−^. In order to get some intuitional understanding of our findings, we further conducted cross-sectional EDS test for control and 0.15% Ca(CF_3_SO_3_)_2_-doped devices before and after applying 1 V reverse bias for 100 s. As shown in Fig. S11, before applying the reverse bias both the control and 0.15% Ca(CF_3_SO_3_)_2_-doped device show identical distribution of Pb and I elements. After aging, the iodide ions in the control perovskite layer show obvious downward shift to the FTO electrode with reference to the immobile Pb cations, while the iodide migration is to a large degree inhibited in the doped perovskite film. To investigate and decouple the effects of Ca^2+^ and CF_3_SO_3_^−^ ions on the operational stability of PSCs, we further tested the efficiency decay of control, FACF_3_SO_3_ processed and Ca(CF_3_SO_3_)_2_-doped PSCs under continuous one sun equivalent white light-emitting diodes (LED) in N_2_ atmosphere using maximum power point tracking (MPPT). As shown in Fig. 5k, 0.3% FACF_3_SO_3_ processed and control devices retained 70% and 55% of their initial efficiency, respectively. The slightly improved operational stability of FACF_3_SO_3_ processed devices can be attributed to less ion migration pathways and metallic Pb degradation sites due to effective passivation of CF_3_SO_3_^−^ [13, 39]. Compared to FACF_3_SO_3_ processed counterparts, 0.15% Ca(CF_3_SO_3_)_2_-doped device showed a much enhanced stability by retaining 95% of its initial efficiency under continuous illumination for 1000 h, which can be attributed to the further inhibited ion migration owing to the incorporation of Ca^2+^ cations. With the synergistic contribution of CF_3_SO_3_^−^ anions and Ca^2+^ cations, Ca(CF_3_SO_3_)_2_-doped devices have less PbI_2_ residue, deep level defect states and suppressed halide migration, thus acquired a much extended life span.

## Conclusions

In summary, we reported for the first time a special group of sulfonic acid salts that are capable of doping divalent metal cations into perovskite lattice in contrast to its widely used metal halide (such as CaI_2_, BaI_2_) counterparts. Through detailed characterizations of perovskite films, we further verified the crystallization regulation and defect passivation effect of CF_3_SO_3_^−^ anions. Having formed coordination bonds with Pb, perovskite film with Ca(CF_3_SO_3_)_2_ shows less PbI_2_ residue and metallic Pb^0^ defects. To further gain in-depth understanding of doped Ca^2+^ ions, we introduce FACF_3_SO_3_ into precursor and compared the properties of perovskite films with Ca(CF_3_SO_3_)_2_ and FACF_3_SO_3_. The results show that Ca^2+^ ions have ignorable effect on defect density of states in perovskite but significantly suppress halide migration with an enlarged activation energy of 1.246 eV. By combing the positive functions of anions on efficiency and cations on operational stability, we proposed an effective strategy to fabricate efficient and stable perovskite photovoltaic devices. Our work successfully decouples the effects of cations and anions, with which the impact of different doping cations can be clearly evaluated.

## Supplementary Information

Below is the link to the electronic supplementary material.

Supplementary file1 (DOCX 5434 kb)
