# Supplementary material for: Understanding the Decoupled Effects of Cations and Anions Doping for High-Performance Perovskite Solar Cells
Source: Nanomicro Lett. 2025 Feb 14;17:145. doi: 10.1007/s40820-025-01655-x (PMC11828769; doi:10.1007/s40820-025-01655-x)
Supplement: Supplementary file 1 — Supplementary file1 (DOCX 5434 kb) [file 40820_2025_1655_MOESM1_ESM.docx]

Supporting Information for

**Understanding the Decoupled Effects of Cations and Anions Doping for High Performance Perovskite Solar Cells**

Tianxiang Hu^1^, Yixi Wang^3^, Kai Liu^1^, Jia Liu^4^, Haoyang Zhang^1^, Qudrat Ullah Khan^5^, Shijie Dai^1^, Weifan Qian^1^, Ruochen Liu^3^, Yanyan Wang^1^, Chongyuan Li^1^, Zhenru Zhang^1^, Mingxiang Luo^1^, Xiaofei Yue^1^, Chunxiao Cong^1^, Yuan Yongbo^4^, Anran Yu^1^*, Jia Zhang^2^*, Yiqiang Zhan^1,2^*

^1^ Center of Micro-Nano System, School of Information Science and Technology, Fudan University, Shanghai 200438, People’s Republic of China

^2^ The State Key Laboratory of Photovoltaic Science and Technology, Institute of Optoelectronics, Fudan University, Shanghai 200438, People’s Republic of China

^3^ Institute for Electric Light Sources, School of Information Science and Technology, Fudan University, Shanghai 200438, People’s Republic of China

^4^ Hunan Key Laboratory of Nanophotonics and Devices, School of Physics, Central South University, Changsha, Hunan 410083, People’s Republic of China

^5^ Vanced materials Technology (Zhongshan) Co., Ltd. Guangdong, 528437, People Republic China

*Corresponding authors. E-mail: [jia.zhang0219@gmail.com](mailto:jia.zhang0219@gmail.com) (Jia Zhang); [aryu@fudan.edu.cn](mailto:aryu@fudan.edu.cn) (Anran Yu); [yqzhan@fudan.edu.cn](mailto:yqzhan@fudan.edu.cn) (Yiqiang Zhan)

**Note S1 Calculation of the Lattice Constants**

To get detailed understanding of the doped perovskite structure, we further calculate the lattice constant of Mg(CF_3_SO_3_)_2_, Ca(CF_3_SO_3_)_2_, Ba(CF_3_SO_3_)_2_ doped perovskite, respectively. We use the peaks with highest intensity to get the lattice constant in consideration of minimizing computational error.

We firstly calculate the interplanar spacing d using the equation:

$$d= \frac{n\lambda}{2sin\theta}$$

Where λ = 1.542 Å (The wavelength of the incident X-ray)

θ = peak position (In radians)

n = 1 (Order of diffraction)

As our doped perovskites keep the cubic structure, the lattice constants can be further calculated with the following equation:

$$a=b=c=d\cdot\sqrt{h^{2}+k^{2}+l^{2}}$$

where (hkl) represents the crystal indices.

**Supplementary Figures and Tables**


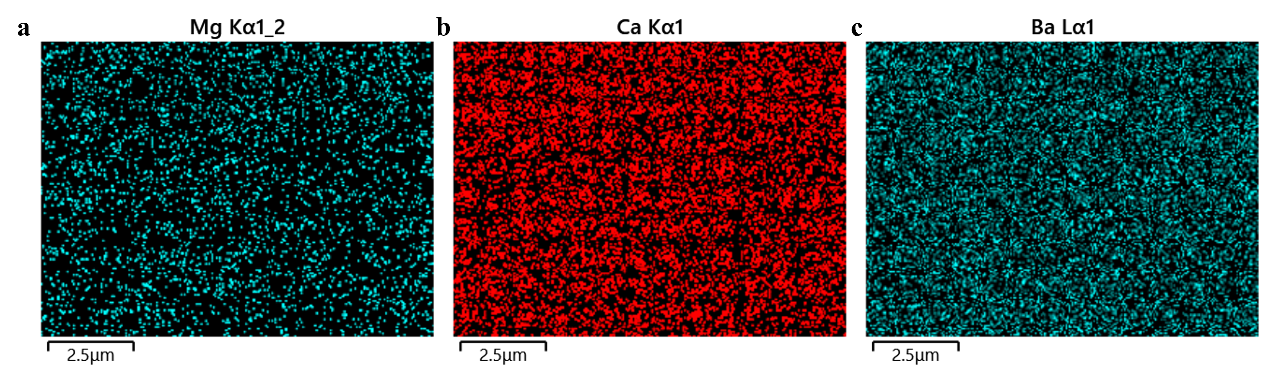


**Fig. S1** The top-surface EDS mapping of (**a**) Mg^2+^ elements in 1% Mg(CF_3_SO_3_)_2_ doped, (**b**) Ca^2+^ elements in 1% Ca(CF_3_SO_3_)_2_ doped and (**c**) Ba^2+^ elements in 1% Ba(CF_3_SO_3_)_2_ doped perovskite films


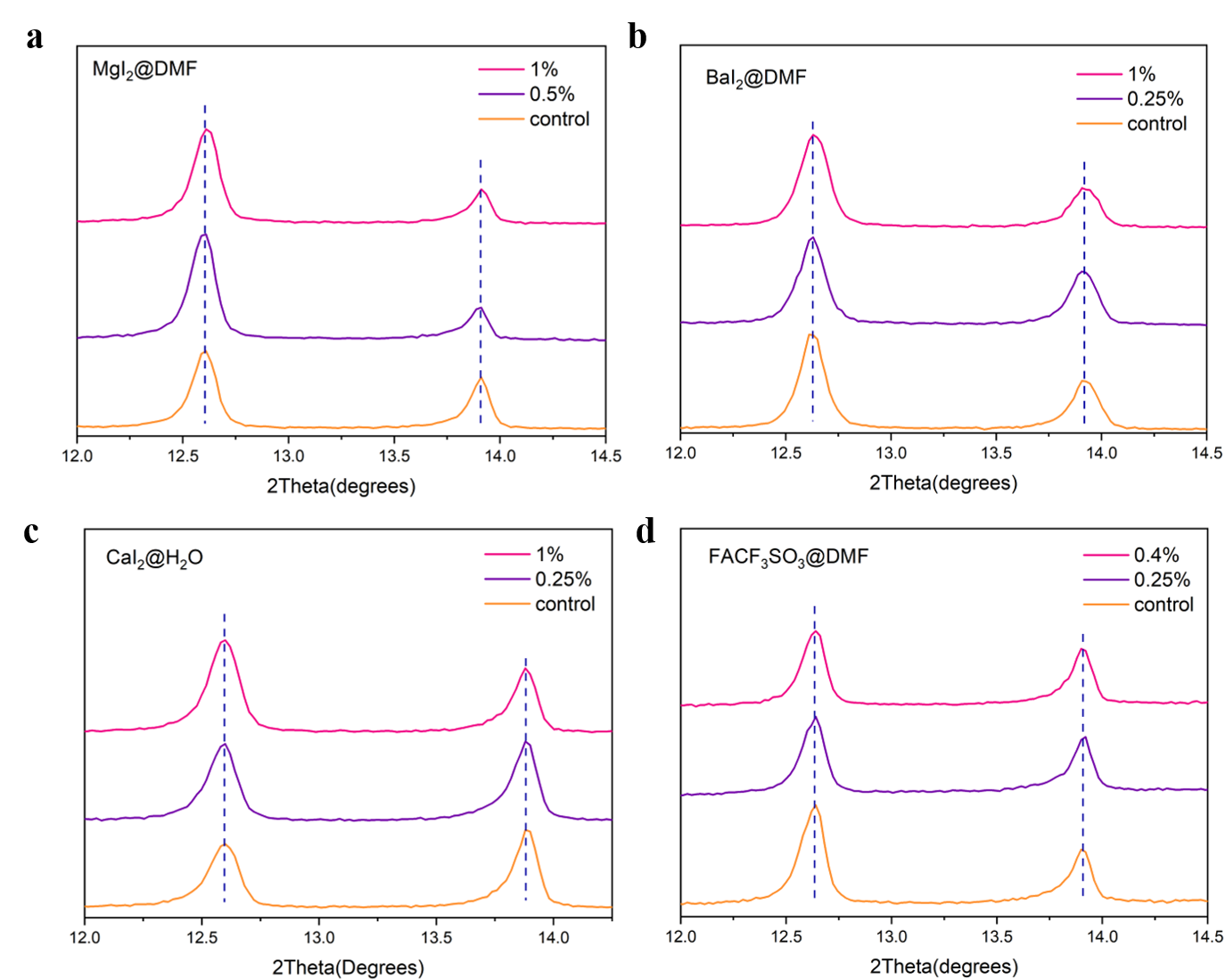


**Fig. S2** XRD pattern of fabricated perovskite film with (**a**) MgI_2_, (**b**) BaI_2_ dissolved in DMF, (**c**) CaI_2_ first dissolved in H_2_O then added to precursor and FACF_3_SO_3_ additive


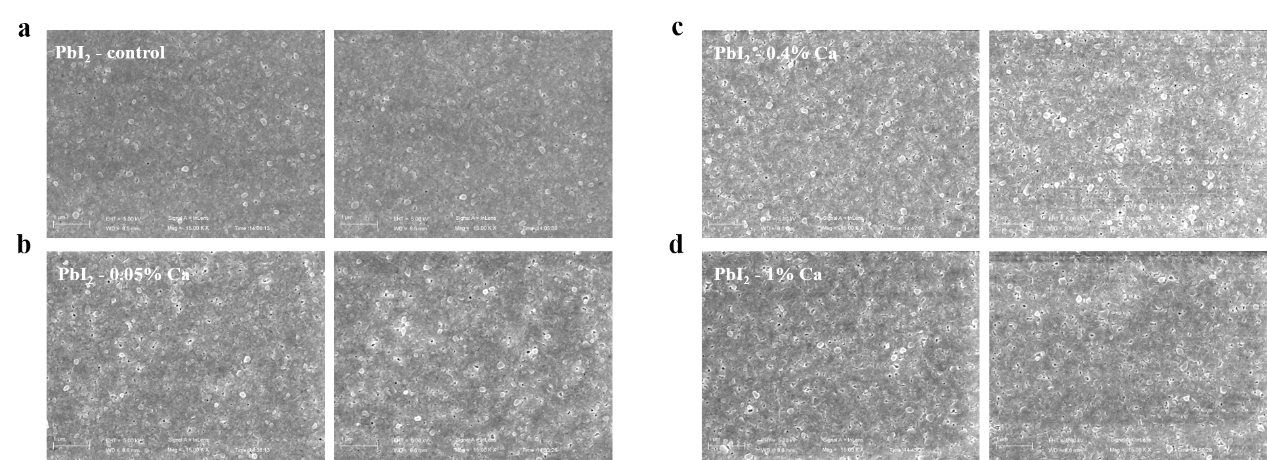


**Fig. S3** Top-view SEM images of (**a**) pristine PbI_2_ film and PbI_2_ film with (**b**) 0.05%, (**c**) 0.4%, (**d**) 1% Ca(CF_3_SO_3_)_2_ dopant


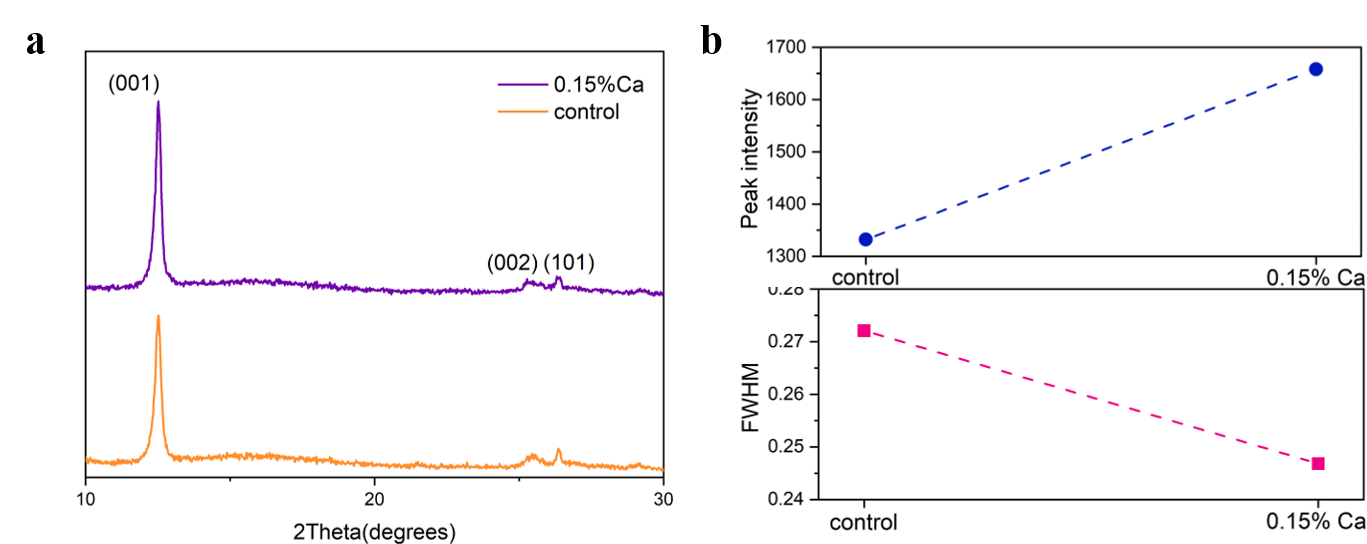


**Fig. S4** (**a**) XRD pattern of control and 0.15% Ca(CF_3_SO_3_)_2_ doped PbI_2_ films. (**b**) Extracted PbI_2_ (001) peak intensity and FWHM from the XRD results


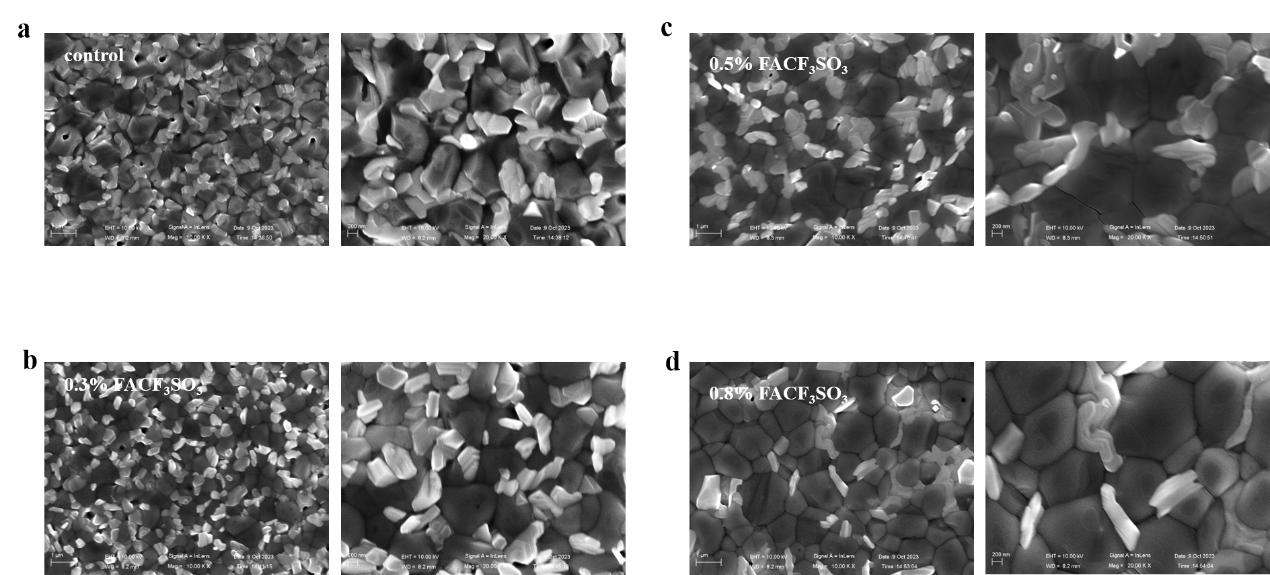


**Fig. S5** Top-view SEM images of (**a**) control perovskite film and perovskite film with (**b**) 0.3%, (**c**) 0.5%, (**d**) 0.8% FACF_3_SO_3_ additive


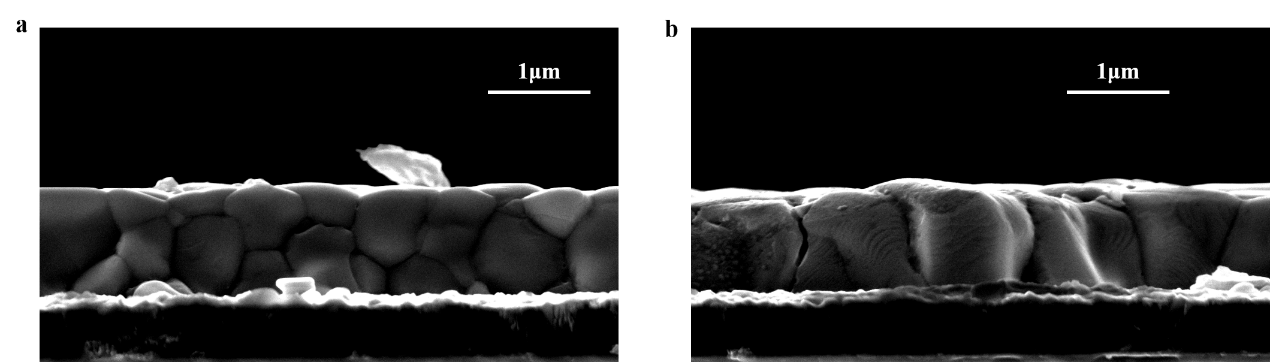


**Fig. S6** Cross-sectional SEM images of (**a**) control perovskite film and (**b**) perovskite film with 0.15% Ca(CF_3_SO_3_)_2_


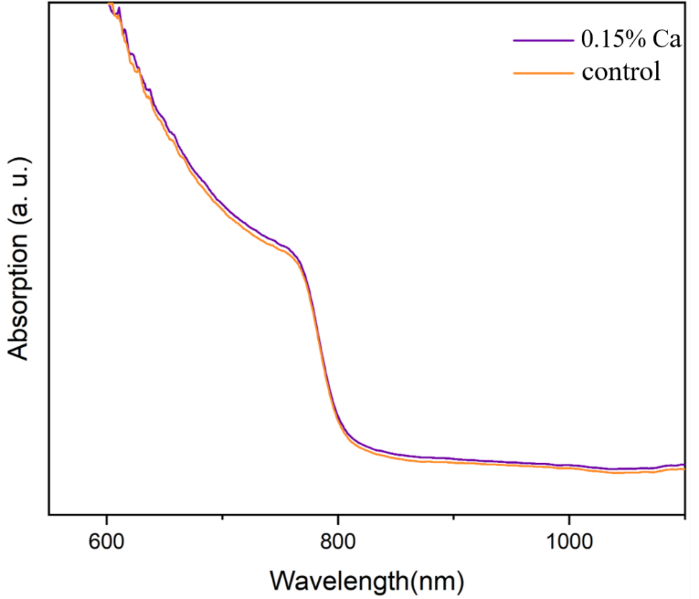


**Fig. S7** UV-vis absorption spectra of perovskite films with and without 0.15% Ca(CF_3_SO_3_)_2_


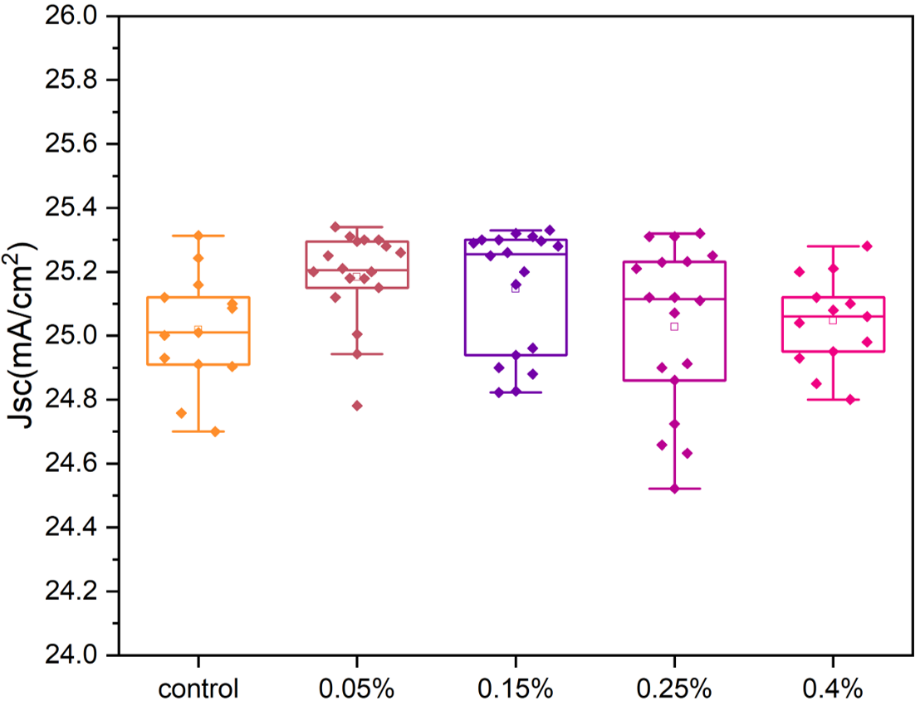


**Fig. S8** Statistical distribution of *J*_SC_ for perovskite solar with cells with different Ca(CF_3_SO_3_)_2_ doping ratio


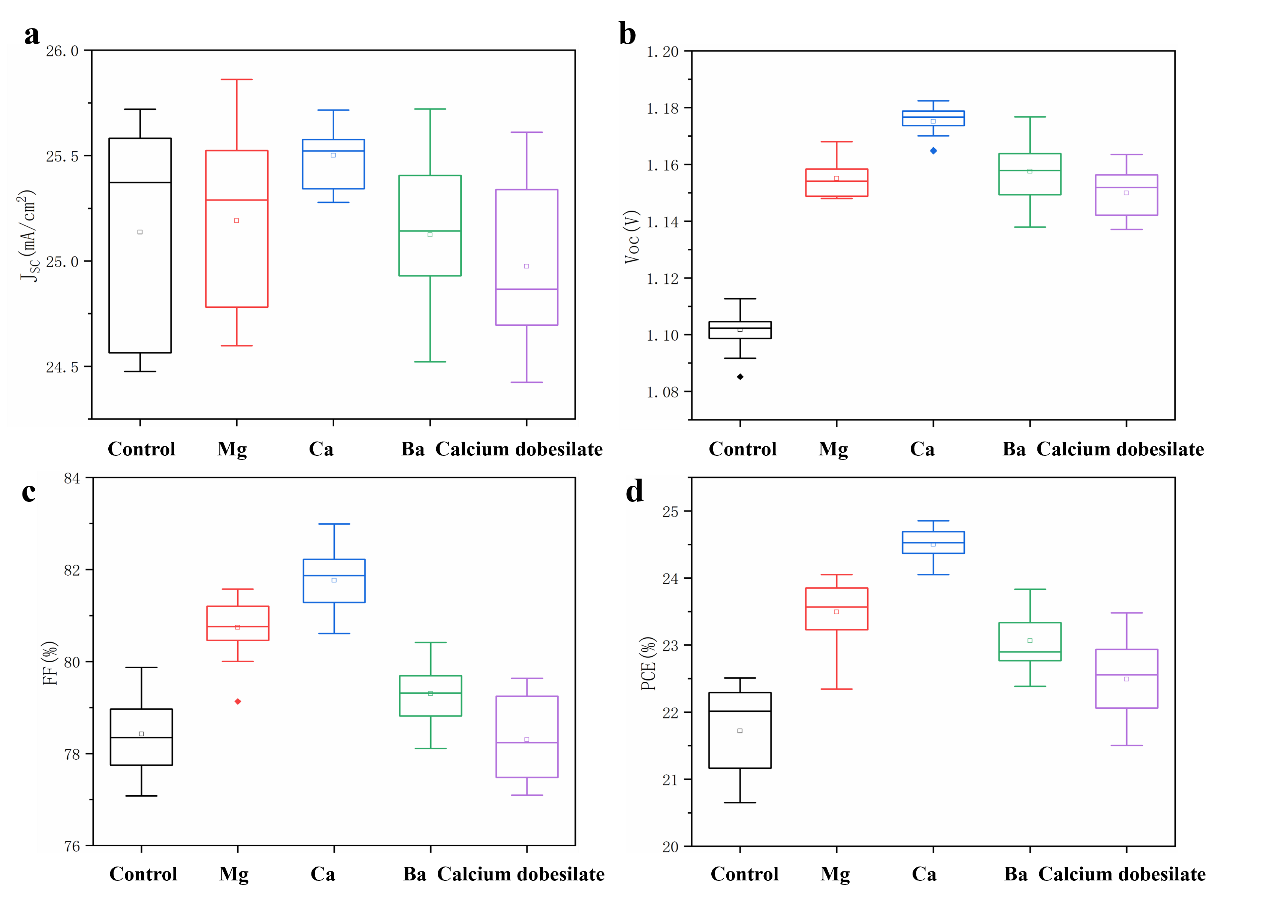


**Fig. S9** Statistical distribution of (**a**) *J*_SC_, (**b**) *V*_OC_, (**c**) FF and (**d**) PCE of control, 0.15% Mg(CF_3_SO_3_)_2_, 0.15% Ca(CF_3_SO_3_)_2_, 0.15% Ba(CF_3_SO_3_)_2_, 0.15% Calcium dobesilate doped devices, respectively


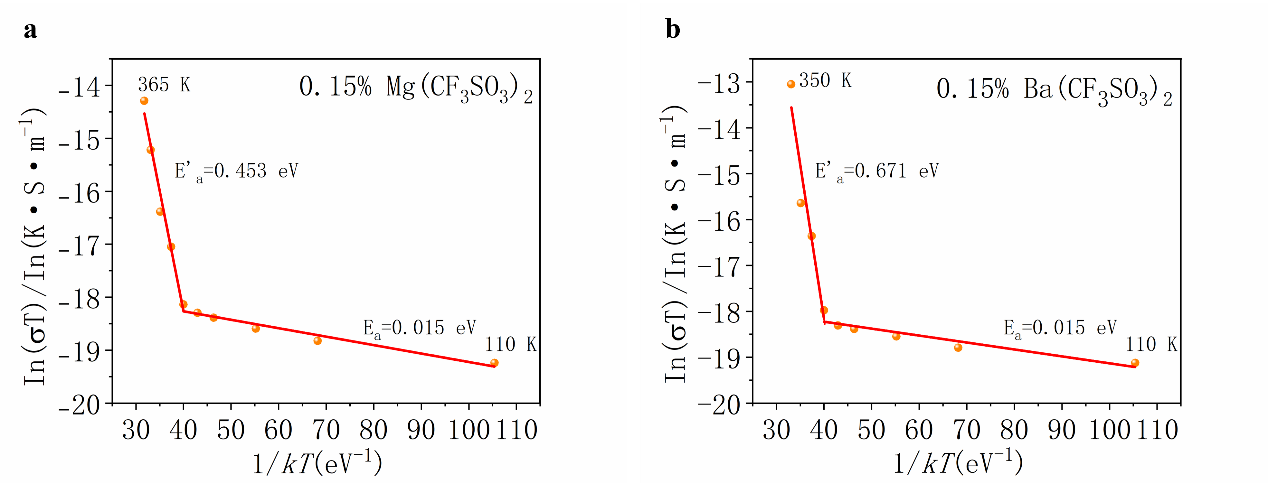


**Fig. S10** Temperature dependent conductivity measurements of lateral devices (Au/perovskite/Au) with (**a**) 0.15% Mg(CF_3_SO_3_)_2_ and (**b**) 0.15% Ba(CF_3_SO_3_)_2_


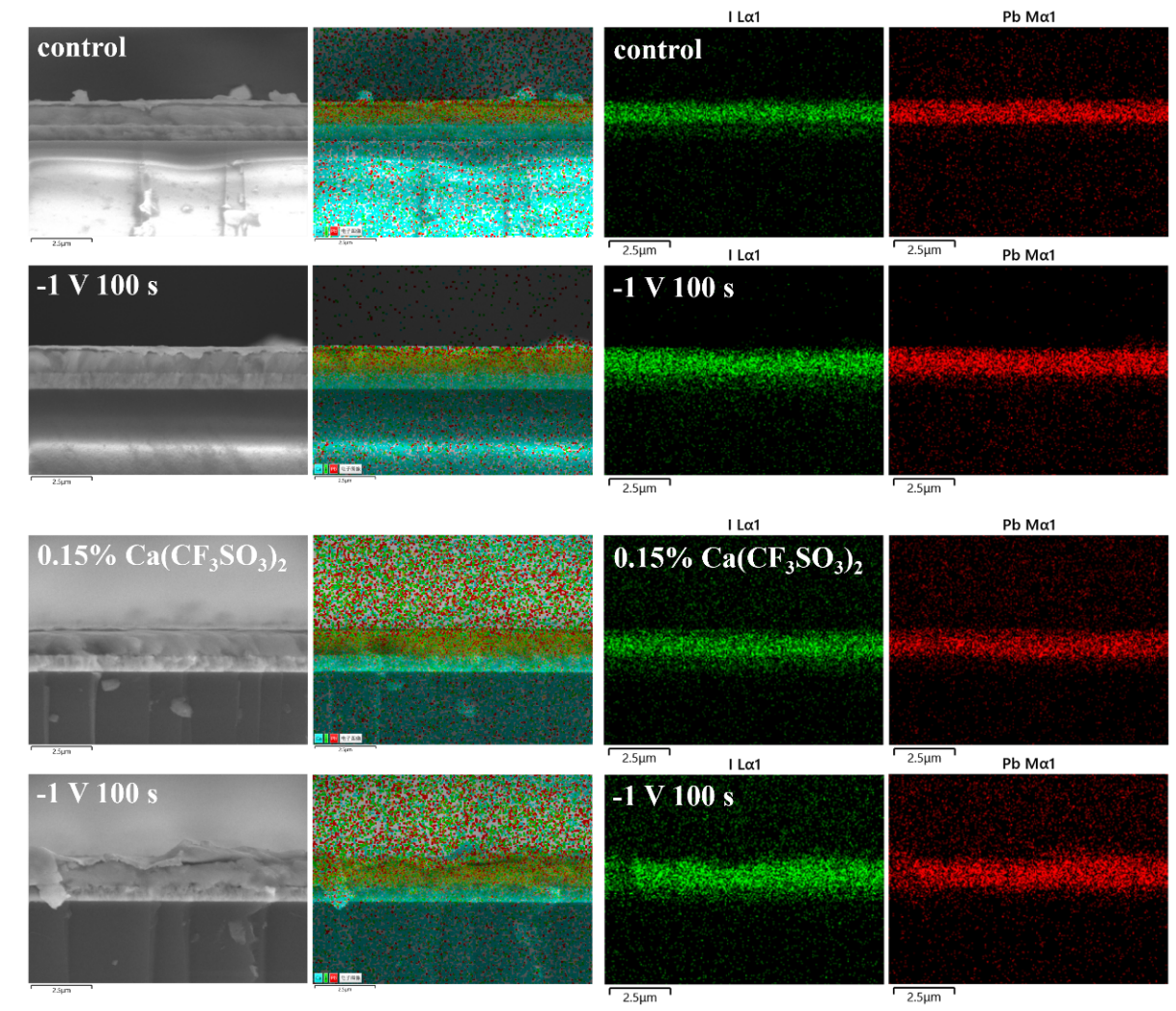


**Fig. S11** Cross-sectional EDS mapping of iodide and Pb elements in perovskite with and without 0.15% Ca(CF_3_SO_3_)_2_

**Table S1** Fitted parameters for the data in Fig. 1h

|  | (001) position | (001) width | (111) position | (111) width |
| --- | --- | --- | --- | --- |
| control | 13.88741 | 0.11417 | 24.24165 | 0.10738 |
| 1% Mg(CF_3_SO_3_)_2_ | 13.86796 | 0.09622 | 24.19939 | 0.10718 |
| 0.4% Ca(CF_3_SO_3_)_2_ | 13.88395 | 0.01157 | 24.21797 | 0.02818 |
| 1% Ba(CF_3_SO_3_)_2_ | 13.8496 | 0.1235 | 24.17839 | 0.14418 |

**Table S2** Calculated lattice constants for control, 1% Mg(CF_3_SO_3_)_2_ doped, 0.4% Ca(CF_3_SO_3_)_2_ doped and 1% Ba(CF_3_SO_3_)_2_ doped perovskites based on the method described in Note S1

|  | From (001) | From (111) |
| --- | --- | --- |
| control | 6.37748 Å | 6.35989 Å |
| 1% Mg(CF_3_SO_3_)_2_ | 6.38638 Å | 6.37083 Å |
| 0.4% Ca(CF_3_SO_3_)_2_ | 6.37907 Å | 6.36602 Å |
| 1% Ba(CF_3_SO_3_)_2_ | 6.39481 Å | 6.37628 Å |
